# Chopart's Operation Previously Described by Mr. Hey

**Published:** 1816-12

**Authors:** 


*For the London Medical and Physical Journal.*

### *Chopart's Operation previously Described by Mr. Hey;* by AMICUS.

IN reading Mr. Yeatman's case of Amputation at the Instep, in the last Number of your Journal, I was much surprised to find no allusion made to the same operation, as recommended and published, some years ago, by one of the oldest and ablest surgeons in this country. In making this observation, I wish only to claim some merit for my own countryman. And though, on comparison of dates, M. Chopart was the first to recommend this mode of operation, yet that it was also original with Mr. Hey, there can be no doubt, on perusal of his "Surgical Observations."

There is some slight difference in the manner of making the flap; but, when practicable, Mr. Hey's method, in my opinion, is the better, in as much as it affords a firmer cushion, and one far less likely to be injured by blows, to which the foot is so much exposed.

*Nov.* 7, 1816.

---

*For the London Medical and Physical Journal.*

